# Abiraterone acetate and prednisone in metastatic castration-resistant prostate cancer: a real-world retrospective study in China

**DOI:** 10.3389/fendo.2023.1158949

**Published:** 2023-05-12

**Authors:** Min Liu, Jiaqing Yan, Kaidi Le, Ying Li, Nianzeng Xing, Guohui Li

**Affiliations:** ^1^Department of Pharmacy, National Cancer Center/National Clinical Research Center for Cancer/Cancer Hospital, Chinese Academy of Medical Sciences and Peking Union Medical College, Beijing, China; ^2^Department of Urology, National Cancer Center/National Clinical Research Center for Cancer/Cancer Hospital, Chinese Academy of Medical Sciences and Peking Union Medical College, Beijing, China

**Keywords:** abiraterone, castration-resistant prostate cancer, outcome, progression, PSA

## Abstract

**Background:**

This research work was aimed at evaluating the incidence and risk factors of adverse events (AEs) occurring in patients treated with abiraterone acetate (AA) and prednisone (PDN) outside clinical trials. These associations were assessed regarding the survival outcomes.

**Methods:**

The study included 191 patients aged ≥18 years of confirmed metastatic castration-resistant prostate cancer (mCRPC) between March 2017 and April 2022. AE incidences were descriptively summarized from the whole cohort. Baseline characteristics, safety (treatment-emergent AEs and severe AEs), and efficacy [progression-free survival (PFS)] were analyzed. Multi-variable Cox proportional hazards models were employed to assess the factors linked with PFS.

**Results:**

Overall, the median PFS was 17.16 months (range, 0.5–57.58). Patient baseline prostate-specific antigen (PSA) ≧̸10 ng/ml (*p* = 0.000), multiple organ metastasis (*p* = 0.007), hypertension (*p* = 0.004), and coronary heart disease (*p* = 0.004) were associated with worse PFS; however, radiotherapy (*p* = 0.028) was linked to better PFS at univariate analysis in the overall cohort. Baseline multiple organ metastasis, hypertension, and radiotherapy remained statistically significant in multivariable models (*p* = 0.007, *p*= 0.005, and *p* = 0.011, respectively).Incidence of AEs showed increased bilirubin (BIL) (55/191 patients, 28.8%) followed by increased alanine aminotransferase/aspartate aminotransferase (ALT/AST) (48/191 patients, 25.09%). The most common grade 3 AEs were increased ALT (3/191, 1.57%) followed by elevated BIL, hypercholesterolemia, and hypokalemia. Anemia had shorter PFS. There were no unexpected AEs in any patient.

**Conclusion:**

AA is effective and tolerated in asymptomatic or slightly symptomatic mCRPC in “real-life” setting. The survival outcomes are influenced by multiple organ metastasis, hypertension, and radiotherapy.

## 1 Introduction

Prostate cancer is among the top 3 causes of solid tumors in men regarding incidence and fatality rates in Europe and United States (1). Men’s health is at risk from prostate cancer, especially in older men. An advanced form known as castration-resistant prostate cancer (CRPC) progresses despite testosterone deprivation therapy or surgical castration (2, 3). Imaging evidence of metastatic CRPC (mCRPC) is present in more than 84% prostate tumors, and 10%–20% prostate malignancies are CRPC (3). The mCRPC, in contrast to CRPC, has poor prognosis, shorter lifespan, and no cure yet (3, 4). There have been advancements in treatment options, including paclitaxel chemotherapy, immunotherapy (e.g., sipuleucel-T), radiotherapy (e.g., radium-223), and novel hormonal treatments (e.g., enzalutamide and abiraterone) (5). Depending on tumor amount, these therapies have extended the median overall life of patients with mCRPC (from 9 to 18 months in 2010 to 16 to 35 months in 2016) (3, 6, 7).

AA is a new antiandrogen that selectively inhibits cytochrome P450 17α hydroxylase/17,20-lyase (CYP17). CYP17 is an enzyme of androgen synthesis in cholesterol metabolic pathway, and AA prevents the androgen synthesis in tumor cells, adrenal gland, and testis. AA is thus vital for halting the evolution of prostate cancer whether used as primary or secondary treatment for mCRPC (2, 3, 8). AA improves antitumor efficiency and safety in clinical trials. It provides survival advantage to mCRPC patients and to mCRPC patients who have not undergone chemotherapy (post-chemotherapy). AA has thus been recommended as standard treatment of mCRPC (4, 5, 9).

Clinical studies have confirmed the effectiveness of AA for treating mCRPC in controlled settings. It is imperative to implement these therapies in actual practice for assessing the impact on patient outcomes. There is limited evidence of related cure outcomes even though AA has been utilized for a number of years (5, 10). Patients’ data from chemotherapy in four European countries are studied for mCRPC patients receiving AA treatment (11). Time to cure failure (TTF) is described for mCRPC patients treated with AA + prednisone or specific corticosteroids (AAP) prior to chemotherapy, and the impact of baseline features on TTF and PFS is also explored. The higher the alkaline phosphatase (ALP), prostate-specific antigen (PSA), or Eastern Cooperative Oncology Group Performance Status (ECOG-PS) rating at the beginning of AAP, the shorter the TTF, the higher the PSA, or the worse the ECOG-PS rating. TTF and time to progression were longer in patients whose ADT response lasted longer (12 months) (*p* <0.0001). This European study offered insight regarding the characteristics, course, and outcomes of chemotherapy administered to mCRPC patients who received AAP in standard medical practice. Even though patients had less favorable clinical characteristics at the start of treatment than those observed in COU-AA-02 trial group, the therapeutic efficacy of AAP was maintained. Another medical experience had demonstrated that OS after AA in patients who underwent chemotherapy (18.1 months) was shorter in clinical practice outside the trial setting than that reported in COU-AA-02 trial (34.7 months), and OS was especially shorter in patients with visceral metastases (2.8 months) (12). National Medical Products Administration (NMPA) approved abiraterone acetate (AA) in 2015 for treating mCRPC. Prostate cancer diagnosis rates, disease makeup, malignancy, and overall effectiveness vary between China, Europe, and America. Most prostate cancer patients in China are diagnosed at advanced stage (13, 14). To support the current clinical therapies, the discrepancy between real treatment status of Chinese mCRPC patients and that stated in the literature needs assessment. The safety and efficacy of AA response may depend on patients’ preexisting comorbidities, which have not been explored in Chinese population. The current study assessed the real-world treatment duration using data from the National Cancer Center and looked at the outcomes in patients with comorbid conditions like hypertension, coronary heart disease, arrhythmias, and diabetes, evaluating the incidence and risk factors of adverse events (AEs) occurring in patients treated with abiraterone acetate (AA) and prednisone (PDN) outside clinical trials.

## 2 Patients and methods

### 2.1 Ethics statement

According to the “authorization 9 September 2016—general authorization for the handling of personal data for scientific research purposes—15 December 2016” (No. 303 of the Milano bulletin published on 29 December 2016), the ethical review and subsequent informed consent were not necessary for this study. Universities, research facilities, and scientific organizations can perform observational studies on previously collected data based on this authorization without getting ethical review or having consequences for the patients involved.

### 2.2 Patient selection

This was a retrospective observational cohort study of 191 mCRPC patients enrolled at Chinese Cancer Center. Data collection period for each patient ranged from initial treatment at prostate cancer diagnosis to 31 October 2022. The mCRPC patients were defined based on exposure to ≥1 of NMPA-approved mCRPC drugs (estramustine, docetaxel, and abiraterone acetate).

Data for abiraterone initial treatment were considered as baseline. Data were extracted from medical records of eligible patients treated with AA and entered in an electronic case report form (eCRF). Patients included in the study orally received 1,000 mg AA once daily and 5 mg PDN orally as twice daily until disease progression, worsening of symptoms, or unacceptable toxicity. All patients were on concurrent androgen deprivation therapy. The incidence of adverse effects including fluid retention, hypertension, hypokalemia, cardiovascular (CV) disease, aspartate transaminase (AST)/alanine transaminase (ALT) elevation, diabetes mellitus, and hypercholesterolemia were annotated and scored using the common terminology criteria for adverse events version 5.0 (15).

### 2.3 Data collection and results evaluation

The hospital information system was used to retrieve the patients’ computerized clinical records. The Prostate Cancer Clinical Trials Working Group (PCWG-2) criteria were employed to characterize the clinically, biochemically, and radiographically progressing illness (16).

Demographic and clinical data were obtained at the time of AAP therapy commencement and termination. Age, comorbidities, biochemical indicators, baseline prostate-specific antigen (PSA), hemoglobin, albumin, AST, ALT, creatinine, ECOG-PS, and Gleason score were also considered.

Primary outcome of the trial was PSA response (PSA50), defined as a decrease >50% compared to baseline. Secondary objectives included progression-free survival(PFS). PFS was defined as the time from commencement of AAP treatment to treatment withdrawal for reasons including disease progression, intolerance to treatment, or death and was deemed as treatment duration. Data on therapies were also collected after the termination of AAP.

Time from the beginning of AAP treatment to progression or death was declared as PFS. The following points were evaluated by a doctor to determine the progression (information from the medical record about normal care): radiographic progression, PSA progression, symptomatic/clinical progression, or radiographic evidence of new metastasis and/or tumor spreading as measured through bone, computer tomography (CT), and/or magnetic resonance imaging (MRI) scans (patient record confirmed one or more of the following: increased pain, skeletal events, increased requirement for analgesics, or palliative radiotherapy). Doctor made the above-mentioned evaluations to determine advancement. Pathological fractures, spinal cord compression, radiation, and bone surgery are the skeletal events (17).

The serious or non-serious adverse drug reactions (ADRs) after exposure to AAP were recorded if they had been demonstrated in patients’ computerized clinical records as at least perchance associated to AAP.

Patients were described having PSA flare who had transient serum PSA upsurge, however not to the extent of biochemical development (PCWG-2 criteria) and accompanied by a drop. In treating prostate cancer due to “PSA flare phenomenon,” some patients at the early stage of therapy may show PSA rise, tumor quantity increase, signs of aggravated or metastatic lesion deterioration, and other ailments (10, 18). The patient may not gain advantages from the cure if remedy is aborted due to “flashing phenomenon” that is ineffective judgment of the treatment. To examine the precise cure efficacy and to take therapy benefits, the clinical trial collaboration team for prostate cancer (PCWG) encouraged 12 weeks as the minimum drug exposure time of first-line drug therapy for CRPC patients (19).

### 2.4 Statistical analysis

Data had been analyzed by SPSS 27.0. Continuous variables were mentioned as mean and standard deviation (SD) or median and range based on their distribution. Variables were compared with one-way ANOVA. Categorical variables were expressed as absolute quantity and percentage as analyzed by the chi-square test. Survival curves had been estimated through product-limit approach of Kaplan–Meier and compared using log-rank statistics. Cox regression mannequin was used to estimate the hazard ratio (HR) and 95% confidence intervals (CI). An alpha fee of 5% was viewed as the threshold for significance.

## 3 Results

### 3.1 Patient characteristics

From March 2017 to April 2022, 191 prostate cancer patients using abiraterone were identified from the hospital information system of Cancer Hospital, Chinese Academy of Medical Sciences. The patient characteristics are summarized in **Table 1**. The median age was 73 years at the time of prostate cancer diagnosis, and nearly half were 75 years old at their first use of mCRPC drug. This was consistent with the other literature reports (2, 8).

**Table 1:** Main characteristic of patients at AA plus PDN start.

	All patients (N = 191)
Median age (years) (range)	73 (49–90)
Age group, n (%)	
≤75 years	98 (51.31%)
>75 years	93 (48.69%)
Time from initial diagnosis to first dose(years)	3.00 (0.00–14)
Time from LHRH to first dose (months)	34.00 (0–168.0)
Preexisting CV disorders, n (%)	
Hypertension	69 (36.12%)
Coronary heart disease	8 (4.19%)
Rhythm disorders	3 (4.05%)
Valvular dysfunctions	2 (1.05%)
Stroke	4 (2.09%)
Preexisting dysmetabolic conditions, n (%)	
Diabetes	37 (19.37%)
Hyperlipidemia	2 (1.05%)
Chronic renal failure	12 (6.28%)
BMI, n (%)	
≤25 (normal weight)	101 (52.69%)
>25 <30 (overweight)	90 (47.12%)
Laboratory parameters, median (IQR)	
Albumin (g/L)	43.00 (40.30–45.20)
Creatinine (μmol/L)	75.00 (64.30–87.75)
Hemoglobin(g/L)	134.00 (124.0–144.0)
ALT(U/L)	16.00 (11.00–21.00)
AST(U/L)	20.00 (16.80–24.00)
PSA(ng/L), median (range)	10.43 (0.003–2876)
Testosterone(ng/L), median (range)	0.0288 (0.025–0.086)
Metastatic sites, n (%)	
Bone only	142 (74.34%)
Pelvic nodes only	19 (9.95%)
Multiple sites (bone + others)	16 (8.38%)
ECOG performance status, n (%)	
<2(0/1)	94 (49.21%)
≥2(2/3/4)	97 (50.79%)
Pain, n (%)	
Absent	127 (66.49%)
Present	64 (33.51%)
Gleason score, n (%)	
⩽7	41(21.47%)
>7	150 (78.53%)

The time between diagnosis and first use of abiraterone was approximately 3 years (range, 0–14), which was shorter than the reported 5.6 years (20). The time from LHRH to first-dose AA was ~34 months, shorter than the reported 46.75 xmonths (21). Most patients had concurrent cardiovascular diseases such as hypertension (69/36.12%), coronary heart disease (8/4.19%), arrhythmia (3/4.05%), and stroke history (4/2.09%), and metabolic diseases like type 2 diabetes (37/19.37%), hyperlipidemia (2/1.05%), and chronic renal failure (12/6.28%). The patients with BMI ≤25 accounted for 52.69%, indicating that the weight of nearly half of the patients was normal. About half were overweight or obese (90/47.12%) before starting AA and PDN. The biochemical indicators of abiraterone treatment were 134.0 g/L hemoglobin (interquartile range (IQR), 124.0–144.0), 75 μmol/L creatinine (IQR, 64.30–87.75), 16.00 U/L ALT (IQR, 11.00–21.00), and 20.00 U/L AST (IQR, 16.80–24.00), and the median of PSA’s treatment initial state was 10.43 ng/L (range, 0.003–2876), and 0.0288 ng/L testosterone (range, 0.025–0.086). When treated with abiraterone, 142/74.34% patients had metastasis of the bone, 19/9.95% of the pelvic lymph node, and 16/8.38% of multiple organs including the bone. A total of 97/50.79% patients had ECOG performance score ≥2, indicating nearly half of the patients with poor condition. Approximately 33.51% of the patients had pain and were treated with analgesic. The patients with a Gleason score >7 accounted for 78.53%, indicating most patients with higher degree of pathological type.

### 3.2 Treatment details

**Table 2** showed that radical prostatectomy was performed in 32/16.70% prostate patients at diagnosis, while radical treatment was 33.8% in some countries (9). The proportion of patients receiving radiotherapy was 6.18%. A total of 173/90.5% cases received ADT, and ADT treatment reported in foreign literature accounted for 40% (9). This can be related to the late disease stage in our patients. The median time from prostate cancer diagnosis to initiation of AA treatment was 3 years (0–14 years); AA treatment duration was 17.16 months, the longest being 57.58 months and minimum being 0.5. Continuation of AA treatment beyond disease progression and post-AA treatments were observed in 34/191 patients, and ADT-treated time before AA was 34 months (0–168 months).

**Table 2:** Treatment details.

	All patients (N =191)
PSA response (%)	124 (64.86)
≥50% PSA decline from baseline	69 (36.1)
≥90% PSA decline from baseline	42 (22.2)
Median time on PSA50 (months)	2.0 (0.5–8.9)
PSA flare (%)	36 (19.0)
Treatment	
Surgery	32 (16.7)
Radiotherapy	50 (6.18)
ADT	173 (90.5)
Time since diagnosis at treatment start (years)	3.0 (0–14)
Treatment duration of AA (months)	17.16 (0.5–57.58)
Median time on ADT in months (range)	34 (0–168)
Reasons of discontinuing AA (%)	
Disease progression	90 (89.11)
Treatment-related complication	8 (7.92)
Patient’s decision	3 (2.97)
Disease progression prior AA (%)	
Biochemical progression only	119 (62.30)
Clinical or radiographic progression	72 (37.70)
LOT1	16 (8.11)
LOT2+	175 (91.89)

LOT1, first line of therapy; LOT2+, beyond second line of therapy.

A total of 101 patients discontinued AA, of which 90/89.11% cases were attributable to disease, 8/7.92% to treatment-related complications, and 3/2.97% to decisions made by themselves. All patients had disease progression prior to AA, 119/62.30% of biochemical progress only, and 72/37.70% due to clinical or imaging progress.

There were 16/8.11% cases who were directly treated with AA as first-line treatment, the proportion of which was lower than other studies (22). This could be due to most patients in this study who had been diagnosed since many years and underwent multi-course therapy. Several patients used abiraterone for nearly 5 years after disease diagnosis and treated with multiple courses.

### 3.3 Outcomes description

#### 3.3.1 Efficacy

##### 3.3.1.1 PSA response

PSA response rate was 64.86%, of which PSA decreased by >50% in 69 cases (36.1%) and >90% in 42 cases (22.2%). The median time of PSA 50 was 2 months with a minimum of 0.5 and maximum of 8.9. A total of 36 patients (19.0%) had PSA flare phenomenon (**Table 2**).

##### 3.3.1.2 Progression-free survival

**Figure 1:**
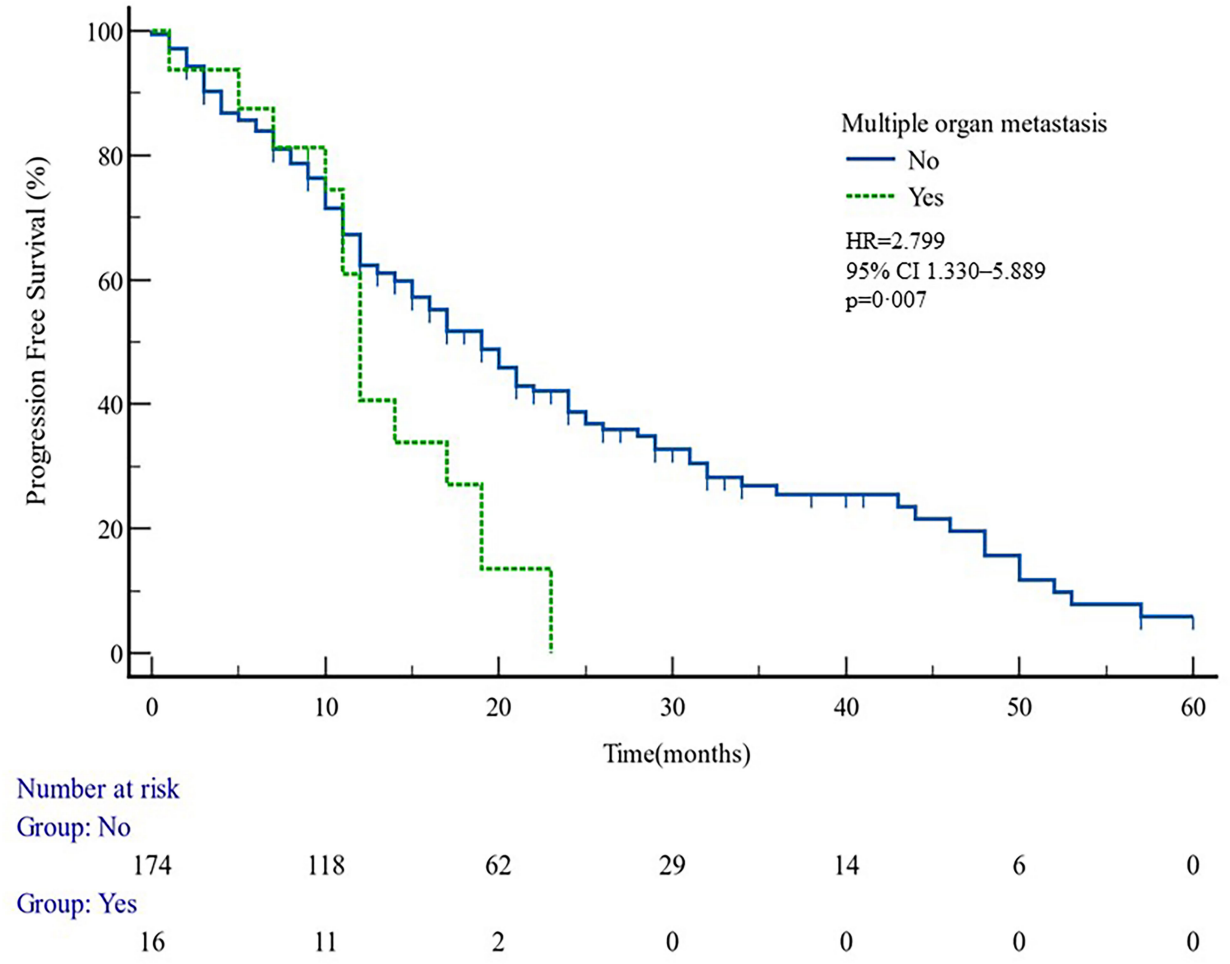
PFS curves in patients with or without multiple organ metastasis. PFS comparison is adjusted for age, PSA value, ECOG-PS, ADT, BMI, and Gleason score.

**Figure 2:**
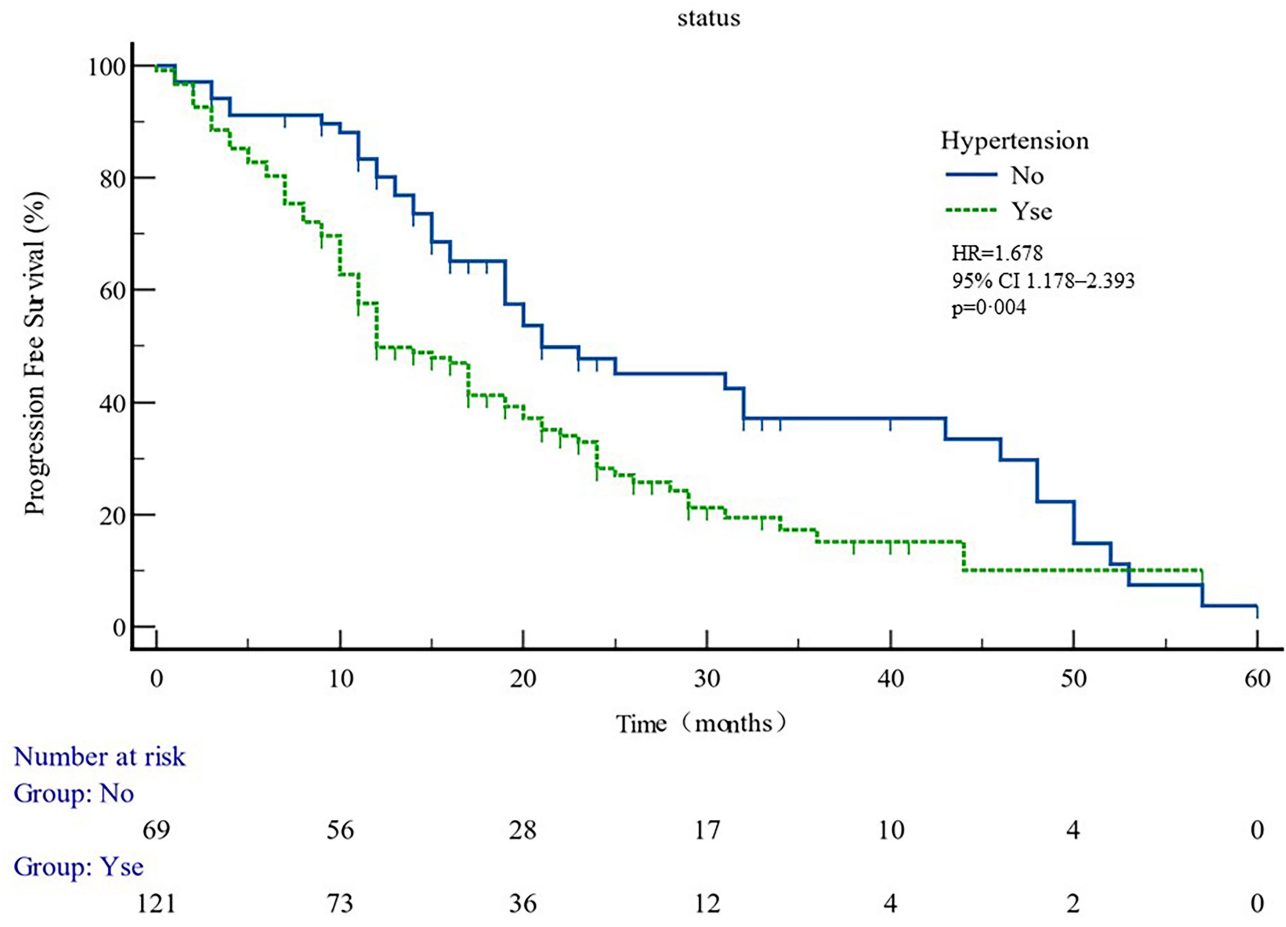
PFS curves in patients with or without hypertension. PFS comparison is adjusted for age, PSA value, ECOG-PS, ADT, BMI, and Gleason score.

**Figure 3:**
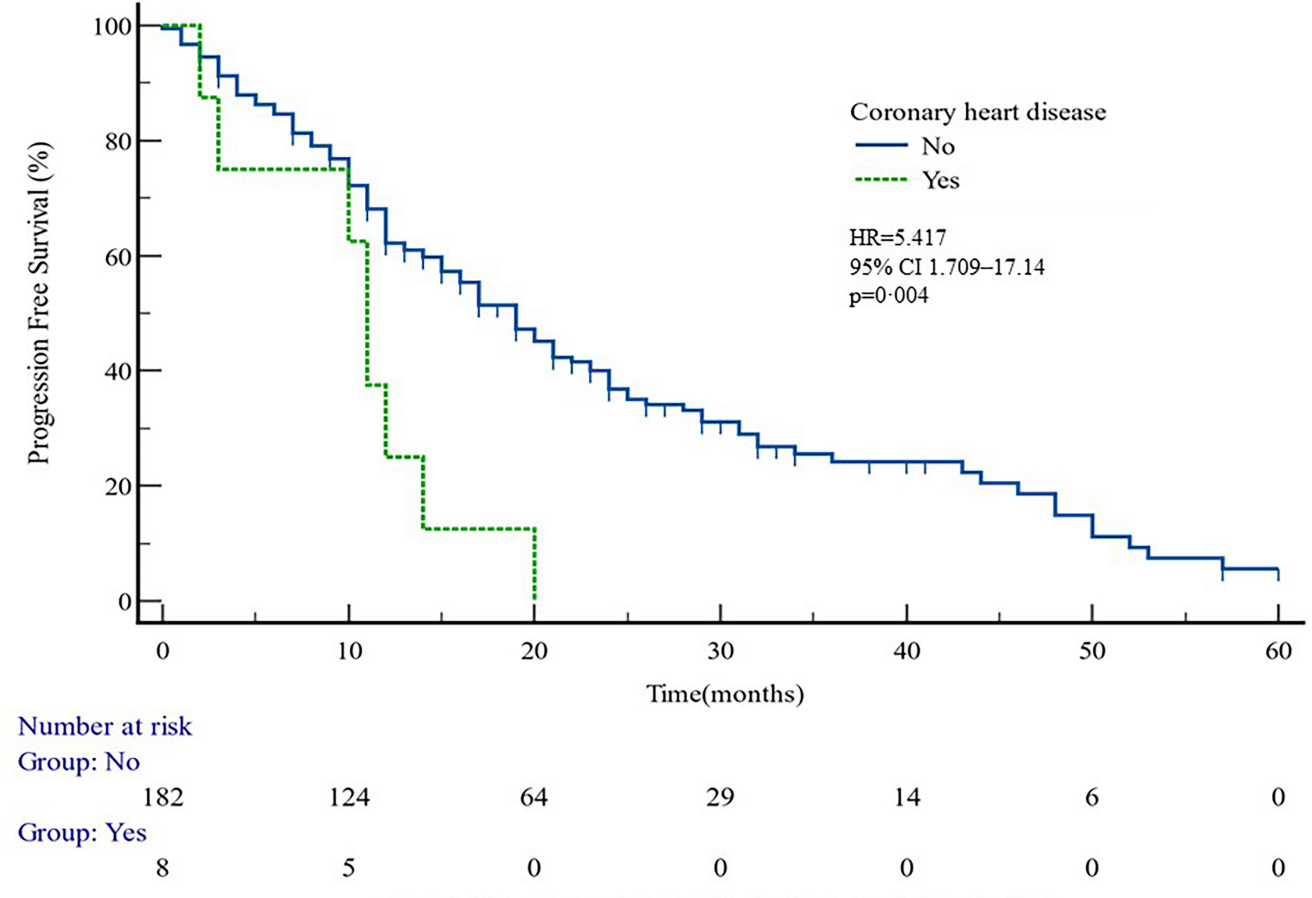
PFS curves in patients with or without coronary heart disease. PFS comparison is adjusted for age, PSA value, ECOG-PS, ADT, BMI, and Gleason score.

**Figure 4:**
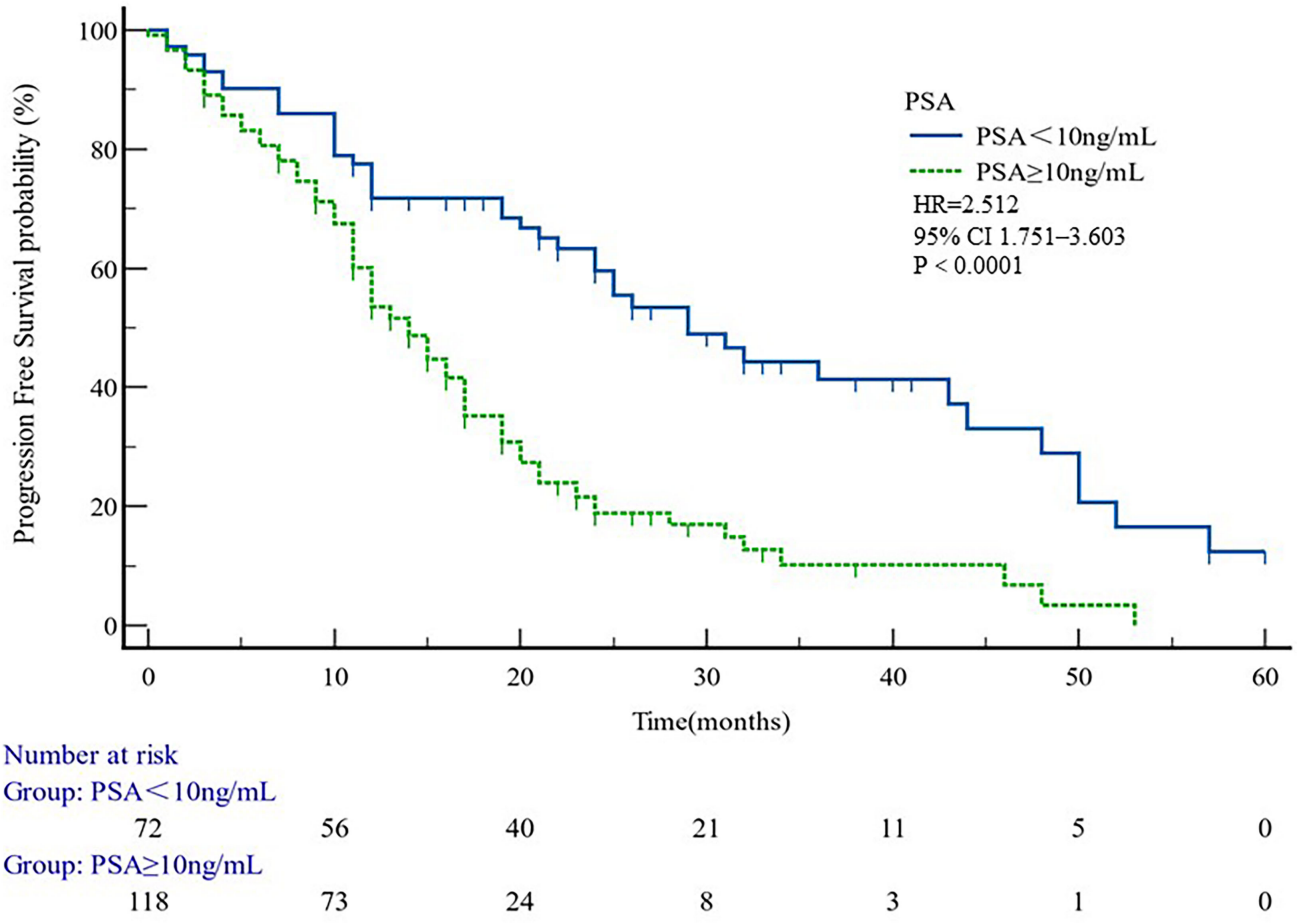
PFS curves in patients PSA < 10 or PSA ≥ 10. PFS comparison is adjusted for age, PSA value, ECOG-PS, ADT, BMI, and Gleason score.

**Figure 5:**
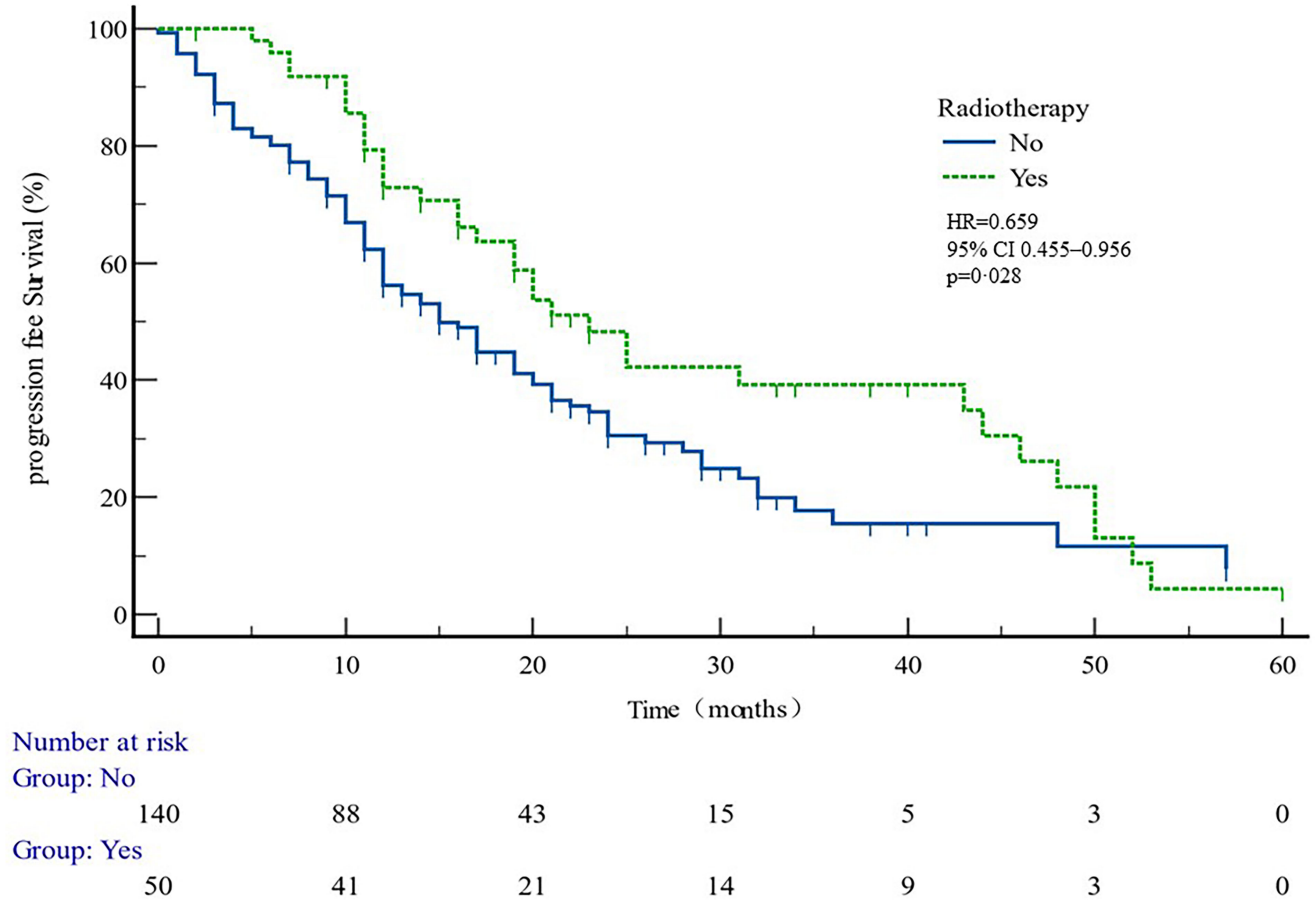
PFS curves in patients with or without radiotherapy. PFS comparison is adjusted for age, PSA value, ECOG-PS, ADT, BMI, and Gleason score.

The median PFS of whole cohort was 17.16 months (range, 0.5–57.58). Patient baseline PSA ≧̸10 ng/ml (*p* = 0.000), multiple organ metastasis (*p* = 0.007), hypertension (*p* = 0.004), coronary heart disease (*p* = 0.004), and radiotherapy (*p* = 0.028) impacted the PFS at univariate analysis of overall cohort. Among these variables, baseline multiple organ metastasis, hypertension, and radiotherapy retained statistical significance in multivariable models (*p* = 0.007, *p* = 0.005, and *p* = 0.011, respectively) (**Table 3**, **Figures 1**–**5**).

**Table 3:** Univariate and multivariate analysis (Cox regression models) assessing the relationship between baseline characteristics and time to treatment failure with AAP.

	Univariate			Multivariate		
	HR	95% CI	p	HR	95% CI	p
Multiple organ metastasis						
Yes	2.799	1.330–5.889	0.007	3.572	1.416–9.068	0.007
Hypertension						
Yes	1.678	1.178–2.393	0.004	2.765	1.362–5.615	0.005
Coronary heart disease						
Yes	5.417	1.709–17.14	0.004	2.226	0.649–7.628	0.203
PSA						
<10 ng/ml	2.512	1.751–3.603	0.000	1.673	0.819–3.416	0.158
Radiotherapy						
Yes	0.659	0.455–0.956	0.028	0.407	0.203–0.815	0.011

#### 3.3.2 Safety data

##### 3.3.2.1 Adverse events

**Table 4** reports the incidence of AEs developed over the course of treatment. A total of 97 patients experienced some level of adverse reactions during treatment. The most common (in ≥10% patients) AE was increased BIL (55/191, 28.8%), followed by increased ALT-AST (48/191, 25.09%). A total of 33 patients developed dyslipidemia. Of the patients, 14 felt tired, and 12 presented hypokalemia. A total of 10 patients had serious cardiovascular adverse reactions characterized by palpitation, arrhythmia, and heart failure. A total of 10 patients developed anemia, and 3 suffered from fluid retention as characterized by weight gain and abdominal swelling pain. One patient was struck with severe vomiting, and patient intolerance resulted in termination of treatment.

**Table 4:** Patients developing at least one AE during treatment.

Graded AEs	n = 191, n (%)	
	Any grade	Grade 3–4
All adverse event	185	11
BIL increased	55 (28.80)	2 (1.05)
ALT-AST increased	48 (25.09)	3 (1.57)
Dyslipidemia	33 (17.28)	2 (1.05)
Fatigue	14 (7.33)	0
Hypokalemia	12 (6.28)	2 (1.05)
Cardiac disorders	10 (5.24)	1 (0.52)
Hyperglycemia	10 (5.24)	1 (0.52)
Anemia	10 (5.24)	0
Vomiting	7 (3.64)	1 (0.52)
Hypertension	6 (3.14)	0
Fluid retention	3 (1.57)	1 (0.52)
Dizziness	3 (1.57)	0
Nausea	3 (1.57)	0
Constipation	2 (1.05)	0
Urinary tract infection	1 (0.52)	0
Skin Rash	1 (0.52)	0
Gastrointestinal bleeding	60	1 (0.52)

AE, adverse event.

Of the patients, 5.24% having diabetes had worsened glucose tolerance, and 17.28% affected by dyslipidemia showed worsening of this condition.

The common grade 3 AEs were increased ALT (3/191, 1.57%) followed by elevated BIL, dyslipidemia, and hypokalemia. Out of the patients with grade 3 liver dysfunction, one needed dose reductions and two needed interruptions due to AEs.

Serious AEs (SAEs) observed through this study were congestive heart failure, fluid retention, hypokalemia, and gastrointestinal bleeding (one patient each, 0.5%).

**Figure 6:**
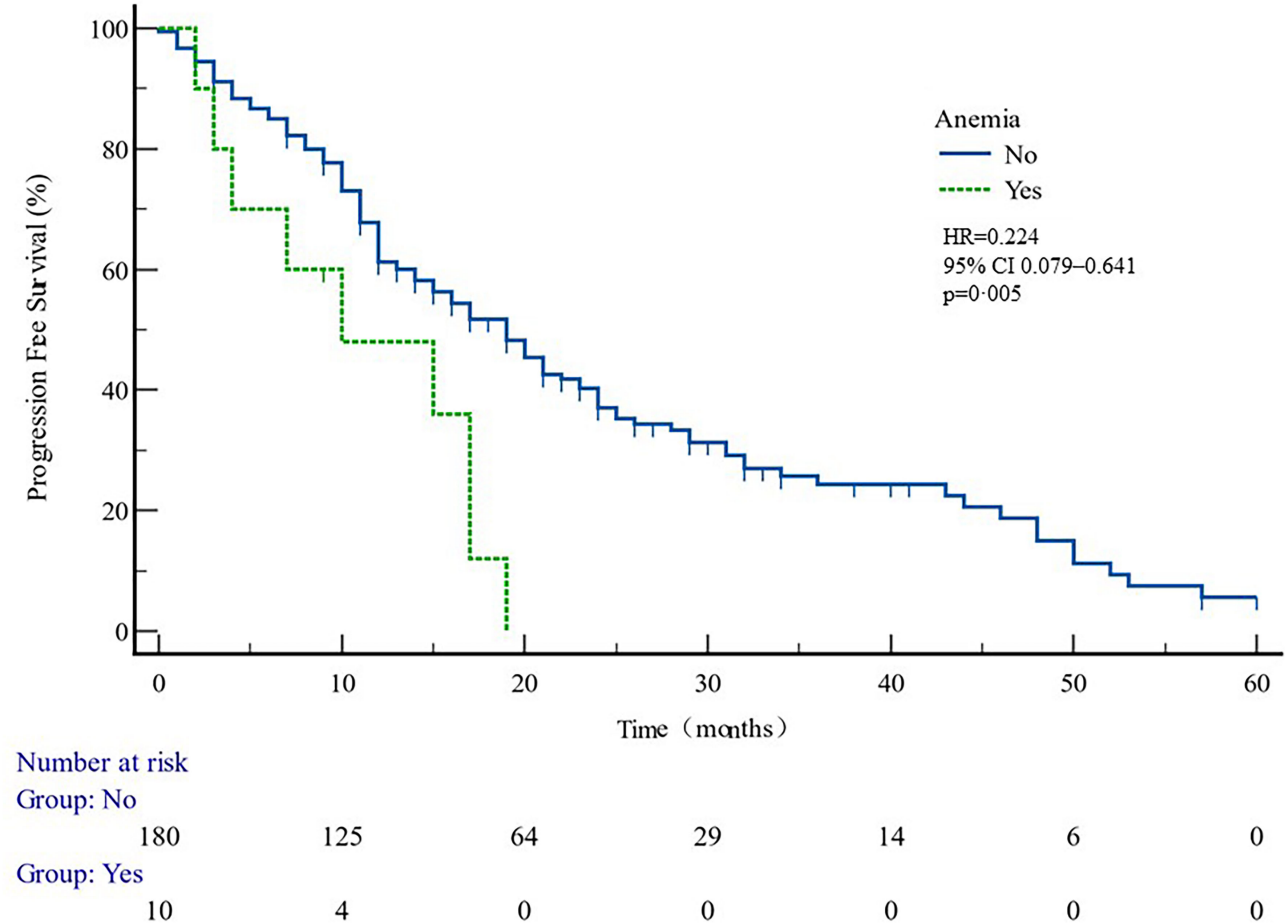
PFS curves in patients with or without anemia ADR. PFS comparison is adjusted for age, PSA value, ECOG-PS, ADT, BMI, and Gleason score.

Patients developing hypokalemia, elevated TBIL, and elevated liver enzymes had better PFS (*p* = 0.040, 0.023, and 0.009 respectively, **Table 5**). Anemia had shorter PFS (*p* = 0.005) (**Figure 6**).

**Table 5:** PFS as a function of the incidence of biochemical events.

AE	PFS		
	HR (95%CI)	p	
Hypokalemia			
No	1.0	
Yes	0.161 (0.028–0.917)	0.040
Elevated TBIL			
No	1.0	
Yes	0.036 (0.002–0.634)	0.023
Elevated liver enzymes			
No	1.0	
Yes	0.060 (0.007–0.491)	0.009
Anemia			
No	1.0	
Yes	0.224 (0.079–0.641)	0.005

AE, adverse event; CI, confidence interval; HR, hazard ratio; PFS, progression-free survival; TBIL, total bilirubin.

## 4 Discussion

Chinese scholars have conducted a series of real-world studies on the survival outcomes of prostate cancer patients in China. A retrospective study evaluated the efficacy and safety of abiraterone acetate in combination with prednisone versus prednisone alone in Asian mCRPC patients who did not receive chemotherapy. After a median follow-up of 14 months, median OS (23.3 vs. 17.5 months), median PSA progression-free survival (Pas-pFs) (10.3 vs. 3 months), and median radiological progression-free survival (rPFS) (13.9 vs. 3.9 months) were significantly higher in the abiraterone group than in the prednisone group (23). A study by Yang et al. (22) was designed to evaluate the safety and quality of life of abiraterone acetate in combination with prednisone in mCRPC patients with cardiovascular disease or risk factors for cardiovascular disease. The study showed that abiraterone acetate did not increase adverse events or decrease quality of life in prostate cancer patients with cardiovascular disease or risk factors for cardiovascular disease. Therefore, such patients should not be excluded from abiraterone acetate therapy. Lin (24) reported that abiraterone plus prednisone was effective in Chinese patients who did not use docetaxel chemotherapy and who were resistant to docetaxel and that lower albumin and visceral metastasis were independent significant predictors of shorter overall survival. An analysis of outcomes in mCRPC patients using Prostate Cancer Registry (PCR) data showed (25) that both first- and second-line AAP therapy were also effective in a large number of mCRPC patients who had not ruled out specific comorbidities. These studies do not shed light on real-world survival risk factors in patients with mCRPC treated with abiraterone. Our retrospective study provided an assessment of the efficacy and safety of AA + PDN in “real world” mCRPC patients and of factors clearly associated with prognosis.

### 4.1 Efficacy data

#### 4.1.1 PSA response

PSA value, which can reflect the tumor load, not only plays an important role in the diagnosis and follow-up of prostate cancer but also is closely related to the prognosis of the disease. The studies of Nayyar (26) and Divrik (27) et al. have confirmed the prognostic factors of metastatic prostate cancer and can predict the response of cancer to drug therapy. PSA is a quick indicator of whether a patient is responding to a drug. Therefore, the guidelines for the clinical management of prostate cancer recommend that PSA be monitored dynamically within 2–3 months after treatment. Many major studies in the field of prostate cancer research have evaluated the effectiveness of treatment in patients with a ≥50% reduction in PSA from baseline after treatment as an indicator of effective PSA response. Our study showed that abiraterone in patients with PSA50 remission rate reached 64.86%, compared with previous studies (20), indicating a better clinical outcome.

#### 4.1.2 Progression-free survival

In patient subgroups of COU-AA-301 and COU-AA-302 trials (11), AA + PDN were superior to PDN and placebo. However, it was not known from the trial data which patients benefitted most from AA and PDN. To find the answer, PFS data was examined in relation to the factors from the past, which predicted clinical outcomes in mCRPC patients (19). After multivariate analysis, our data determined that patients diagnosed with multiple organ metastases and hypertension were associated with shorter PFS, while radiations were associated with longer PFS.

The data suggest that patients with multiple organ metastasis and hypertension have worse outcomes. Radiotherapy is likely to benefit the most from AA plus PDN treatment, independent of age, BMI, ECOG, GS, presence of visceral metastasis, and whether previously treated with DX or not. The short survival of hypertensive patients may be related to the common androgen regulation mechanism of prostate cancer and hypertension, suggesting that the clinical treatment of prostate cancer patients with hypertensive history should be paid attention to. The better clinical outcome of radiotherapy patients may be related to the better baseline situation of these patients.

### 4.2 Safety data

AA plus PDN proved as a well-tolerated regimen among this cohort. Lower incidences of AEs were observed compared with that reported in COU-AA-302 trial (11), and only one episode of grade 3–4 hypokalemia was also observed. Overall, the fluid retention and cardiac disorders were much lower than those recorded in the two pivotal trials (11, 20). Greater incidence of grade 3–4 dyslipidemia, raised BIL, and elevated ALT-AST were detected in 1.05%, 1.57%, and 1.05% of patients, respectively. Overall, this series had reduced fluid retention and cardiac problems compared to two pivotal studies.

This study showed that anemia was associated with shorter PFS (**Figure 6**, **Table 5**). It can be speculated that anemia may act as a predictor of therapeutic effect. It has been reported that pre-treatment anemia is a prognostic factor for mHSPC patients treated with first-line ABI (28). Poor clinical outcomes in anemia patients may be related to tumor hypoxia. Studies have confirmed that hypoxia-inducible factor 1α (HIF-1α) may reduce tumor control by inducing hypoxia (29, 30). HIF-1α activates HIF-1β to form a transcription factor complex, which regulates the expression of multiple genes such as vascular endothelial growth factor and glucose transporter, which are closely associated with angiogenesis and tumor growth (31, 32). Tumor hypoxia is also often related to treatment resistance of other tumors, and hypoxia of tumor PC cells is associated with clinical staging and biochemical recurrence of locally advanced prostate cancer (33). Therefore, it is a key problem for clinicians to correct anemia actively and improve the prognosis of prostate cancer.

## 5 Conclusion

These results demonstrate that AA plus PDN is a safe and effective regimen for all patients including senior people. The incidence of adverse events (AEs), particularly hepatic damage and hypertension, was lower than the numbers reported in pivotal trials. There was no evidence that AEs might indicate worse clinical outcomes; however, it was advisable to choose right patients and monitor them carefully. Following the multivariate study that controlled for patient age, visceral metastasis, and whether the patient had previously received DX, it was determined that multiple organ metastasis and hypertension were related with shorter PFS and that radiations were linked to longer PFS.

People with treatment-related anemia had worse results than those who do not. However, larger trials are needed to corroborate this finding. To help mCRPC patients for better decisions and follow care patterns, studies must be conducted on more precise markers that can predict efficacy and safety of AA and PDN.

## Data availability statement

The raw data supporting the conclusions of this article will be made available by the authors, without undue reservation.

## Ethics statement

The studies involving human participants were reviewed and approved by National Cancer Center/National Clinical Research Center for Cancer/Chinese Academy of Medical Sciences and Peking Union Medical College National GCP Center for Anticancer Drugs, The Independent Ethics Committee. The patients/participants provided their written informed consent to participate in this study.

## Author contributions

ML, NX, and GL were responsible for the comprehensive study design, paper revision, and submission. JY and KL contributed to article search and data extraction. ML and YL performed statistical analysis. All authors contributed to the article and approved the submitted version.
